# Recurrent Urosepsis and Cardiogenic Shock in an Elderly Patient with Pheochromocytoma

**DOI:** 10.1155/2011/759523

**Published:** 2011-09-06

**Authors:** Joan Joo-Ching Khoo, Vanessa Shu-Chuan Au, Richard Yuan-Tud Chen

**Affiliations:** Department of Endocrinology, Changi General Hospital, Singapore 529889

## Abstract

Pheochromocytomas are thought to be uncommon in the elderly. However, the prevalence is likely to be higher than reported, as older patients are less likely to be diagnosed due to absence of classical symptoms of sympathetic overactivity and confounding effects of aging, comorbidities, and medications. We describe a hypertensive elderly patient with incidentally diagnosed pheochromocytoma complicated by recurrent urosepsis, cardiomyopathy, and fatal myocardial infarction. Our case demonstrates that, in older hypertensive patients without classical symptoms, orthostatic hypotension and urinary retention, which are common in the elderly, may indicate catecholamine excess and that the deleterious cardiovascular consequences of catecholamine excess in the elderly are not prevented by pharmacological *α*- and *β*-blockade.

## 1. Introduction

Pheochromocytomas are rare catecholamine-producing tumors of chromaffin cells in the adrenal medulla or extra-adrenal paraganglia. They are diagnosed most frequently in the fourth and fifth decades [1] and are thought to be uncommon in patients aged above 60. Older patients with pheochromocytoma are less likely to experience symptoms of catecholamine excess such as headache, sweating, and palpitations and are, therefore, more likely to be diagnosed incidentally on autopsy [1]. We describe an elderly patient with orthostatic hypotension and urinary retention, who was incidentally diagnosed with pheochromocytoma.

## 2. Case Report

A 77-year-old woman with a history of hypertension on nifedipine LA 30 mg daily, osteoarthritis, and cognitive impairment, who required minimal assistance in activities of daily living, was admitted with fever, postural dizziness, dysuria, and inability to pass urine for one day. Her temperature was 38.9 degrees Celsius, blood pressure (BP) 180/100 mmHg, pulse rate 104/minute, and physical examination revealed a tender distended bladder which drained 2700 mL of turbid urine. She had experienced two episodes of urine retention and incontinence over the previous month, the most recent of which required urethral catheterization and outpatient antibiotic treatment for *E. coli* urinary tract infection (UTI). Urine and blood cultures grew *E. coli*, sensitive to penicillin and ceftriaxone. Her serum creatinine was elevated at 164 *μ*mol/L, which was attributed to dehydration causing prerenal azotemia (urea 8.4 mmol/L), urosepsis, and obstructive uropathy. Serum sodium, potassium, calcium, and phosphate were within normal ranges. Serum urea and creatinine normalized with hydration.

**Figure 1:**
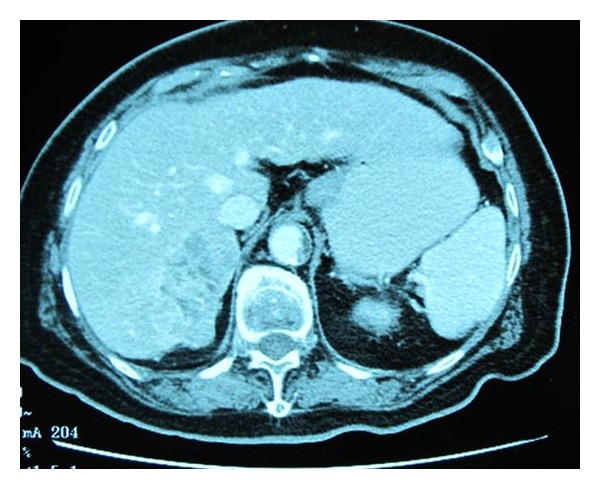
CT scan showing heterogeneous vascular right adrenal mass, suggestive of a pheochromocytoma.

**Figure 2:**
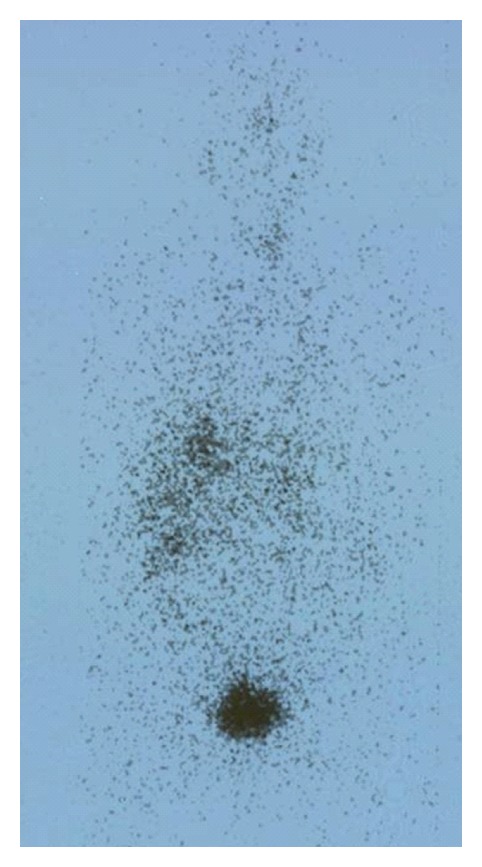
^131^I-MIBG scan showing increased uptake in the right suprarenal region, consistent with adrenal pheochromocytoma.

Her BP was persistently elevated above 170/100 mmHg despite increasing nifedipine LA to 60 mg and adding atenolol. She had postural dizziness and one documented episode of postural hypotension (BP supine 158/72 and standing 106/66 mmHg) on the 3rd day of admission despite intravenous rehydration and clinically normal hydration status. Computed tomography scan of the abdomen, performed to exclude renal abscess, showed a heterogeneous vascular right adrenal mass measuring 6.5 × 3.3 cm that enhanced with contrast, reported as being suggestive of pheochromocytoma (Figure 1). 24-hour urine catecholamine and metanephrine levels on the 5th day of admission were measured: urine adrenaline 28 nmol/day (NR 9.3–122), urine noradrenaline 396 nmol/day (NR 72–505), urine metanephrine 3411 nmol/day (NR 264–1729), urine normetanephrine 5141 nmol/day (NR 480–2424). Random serum cortisol level was 619 nmol/L, 24-hour urine-free cortisol 214 nmol/day, plasma aldosterone 106.2 pmol/L (NR 16.6–617.7), and renin activity was 0.30 *μ*g/L/hr (NR 0.66–3.08). The imaging features of the adrenal mass, and elevation of urine metanephrine and normetanephrine 2-fold above the upper limit of normal, were, therefore, suggestive of pheochromocytoma. A ^131^I-metaiodobenzylguanidine (MIBG) scan demonstrated an MIBG-avid mass in the right suprarenal region, consistent with pheochromocytoma (Figure 2), with no evidence of distant metastases. 24-hour urine-free cortisol and plasma renin and aldosterone were normal. She did not recall having experienced typical symptoms of catecholamine excess such as headache, sweating, or palpitations. Phenoxybenzamine (POB) was started for *α*-adrenergic blockade. Her left ventricular ejection fraction (LVEF) was 45% with grade 1 diastolic dysfunction and left ventricular hypertrophy, but no wall motion abnormalities. Coronary angiogram showed no significant stenosis. As she refused adrenalectomy, she was continued on pharmacological *α*- and *β*-adrenergic blockade. Postural hypotension resolved, and her BP was 130/70 mmHg on POB 10 mg t.d.s. and atenolol 50 mg once daily on discharge.

Over the next 5 years, atenolol was increased to 100 mg daily to maintain BP below 140/90 mmHg. POB dose titration was limited by symptomatic postural hypotension despite clinically adequate hydration. Despite several episodes of acute urinary retention requiring outpatient urethral catheterization, she refused to consider intermittent catherization. She was admitted thrice for urosepsis and during her last admission; intravenous imipenem was started as urine and blood cultures were positive for *Klebsiella pneumoniae* with extended-spectrum betalactamase. She was found to have an acute myocardial infarction with pulmonary oedema on the 3rd day of admission when she went hypotensive and became increasingly dyspnoeic: an electrocardiogram (ECG) showed ST-segment depression and T inversion in the anterior chest leads, and troponin T level was elevated at 0.94 ng/mL (normal range <0.10 ng/mL). *α*- and *β*-blockade were stopped when her BP decreased to 90/52 mmHg, and intravenous frusemide was given cautiously. Echocardiography revealed impaired left ventricular ejection fraction of 20% with biventricular hypokinesia, multiple global wall motion abnormalities, and dilated atria, consistent with cardiomyopathy. Aspirin and subcutaneous fraxiparine were started. Her serum calcium (corrected for albumin) was low at 1.92 mmol/L, which was corrected with intravenous calcium gluconate infusion to 2.14 mmol/L. Shock and acute pulmonary oedema remained intractable despite inotropic support with dopamine infusion, and she became asystolic the same night. Cardio-pulmonary resuscitation was unsuccessful.

## 3. Discussion

Our patient demonstrates that the diagnosis of pheochromocytoma is frequently overlooked in the elderly due to paucity of classical symptoms of sympathetic overactivity, and features of catecholamine excess being confounded by the effects of aging, comorbidities, and medications. In large cohort studies of pheochromocytoma patients, 9–27% were aged 60 or older, but adults in this age group comprised 65–80% of cases diagnosed at autopsy [2]. Pheochromocytoma is thought to be most prevalent in the fourth and fifth decades, with the average age in several large series being *∼*45 years [3–7]. 60–90% of patients in these studies reported at least one of the classic triad of headache, palpitations, and diaphoresis. The lack of classical symptoms in elderly patients increases the difficulty of diagnosing pheochromocytoma, as in our case. Comorbidities such as cerebrovascular and coronary artery disease may confound the clinical features of catecholamine excess. Moreover, aging is associated with decreased baroreceptor function and responsiveness to catecholamines [1]. Common antihypertensives such as betablockers, which our patient was taking, may also obscure the symptoms of sympathetic nervous activation. Unopposed *β*-adrenergic blockade may worsen hypertension without *α*-blockade. Orthostatic hypotension in pheochromocytoma may be due to abnormal sympathetic vascular regulation [8] and increased epinephrine production [1] and causes dizziness and falls which are relatively common and multifactorial in elderly patients, underscoring the need for a high index of suspicion. 

The relatively large size of our patient's tumor may be a cause, as well as a consequence, of the lack of symptoms, which is concordant with observations that pheochromocytomas are more likely to be incidentally diagnosed in older patients [6, 9]. Moreover, as the use of computed tomography scans and magnetic resonance imaging to evaluate abdominal symptoms has become more frequent, the incidence of pheochromocytomas which do not present with classical symptoms has increased [5, 9, 10]. Incidental pheochromocytomas are less likely to be associated with classical symptoms and are larger at diagnosis [5, 10]. Large tumours are paradoxically associated with less intense symptoms and lower catecholamine levels due to catecholamine metabolism within the tumour by catechol-O-methyl-transferase, such that only a small amount of free hormone is released into the circulation despite significantly elevated metanephrine and normetanephrine [11], as was seen in our patient. Moreover, chronically elevated catecholamine levels are associated with downregulation of catecholamine receptors and desensitization to sympathetic stimulation [12]. 

Urinary retention in our patient was likely to be due to the *α*-adrenergic receptors overstimulation in the bladder neck and urethra, seen on urodynamic studies in patients with pheochromocytoma [13], who were found to have abnormally elevated urethral closure pressure which was responsive to treatment with an *α*-adrenergic antagonist. Chronic urine retention increases the risk of urosepsis in elderly patients, in whom urinary tract infections are common due to urinary dysfunction from reduced mobility, comorbidities, and effects of medications. In our patient, urinary retention may have occurred due to suboptimal *α*-adrenergic blockade due to intolerance of postural hypotension. This may have contributed to recurrent urosepsis.

Elderly patients are highly susceptible to the deleterious effects of chronic catecholamine excess and surges of catecholamine secretion, particularly cardiomyopathy and myocardial injury. Catecholamine-induced cardiomyopathy is associated with left ventricular dilatation, dysfunction, and dyskinesia which may be apical (Takotsubos pattern) [14] or basal (inverted Takotsubo) which may be reversible with normalization of catecholamine excess [15]. Our patient had mildly impaired LVEF at diagnosis with no dyskinesia, but during her last episode of urosepsis she had severely reduced LVEF with global dyskinesia, despite absence of significant coronary atherosclerosis five years ago and well-controlled BP on *α*- and *β*-blockade. Poor LVEF was also exacerbated by acute myocardial infarction, which is known to be precipitated by sepsis-induced catecholamine surges [16], leading to norepinephrine-induced increased myocardial oxygen demand (positive chronotropic effect, increased afterload) [15], coronary vasospasm, and directly toxic effects of catecholamines (accelerated myocardial apoptosis and fibrosis by cyclic AMP-mediated intracellular calcium overload, increased lipid mobility, and free radical production [17, 18]). Pulmonary oedema in our patient was multifactorial, due to decompensated left ventricular function from catecholamine cardiomyopathy and acute infarction in the presence of increased peripheral vascular resistance, increased pulmonary venous pressure from overfilling or venoconstriction, and catecholamine-induced hyperpermeability of pulmonary capillaries [15]. Cardiogenic shock was exacerbated by hypocalcemia caused by catecholamine-mediated intracellular sequestration of calcium [19], and possibly, high circulating levels of epinephrine binding to *β*2-adrenoceptors, triggering protein-kinase-A-mediated phosphorylation of the receptor which initiated a switch from Gs to Gi protein signaling [20] called stimulus tracking [21] which further depressed myocardial contractility. Despite the normalization of serum calcium, appropriate antibiotic treatment, and inotropic support, the combination of septic and cardiogenic shock was fatal. Although medical therapy in our patient was effective in controlling hypertension in our patient for five years, it is likely that resection of the pheochromocytoma shortly after diagnosis as recommended [1] would have prevented catecholamine-induced cardiovascular complications. 

## 4. Conclusion

It is important to have a high index of suspicion for pheochromocytoma in hypertensive elderly patients with recurrent urinary retention and orthostatic hypotension. Our patient demonstrates that pharmacological adrenergic blockade does not prevent the potentially fatal cardiovascular complications of pheochromocytoma, supporting the recommendation of adrenalectomy for all patients who are fit for surgery, including the elderly.
